# Supplementary material for: Causal Relationship between Aging and Anorexia Nervosa: A White-Matter-Microstructure-Mediated Mendelian Randomization Analysis
Source: Biomedicines. 2024 Aug 16;12(8):1874. doi: 10.3390/biomedicines12081874 (PMC11351342; doi:10.3390/biomedicines12081874)
Supplement: Supplementary file 1 [file biomedicines-12-01874-s001.zip › Supplementary Figure/Figure S8/Leaveoneout plot of MR analysis for TL to ubm-b-1489.pdf]

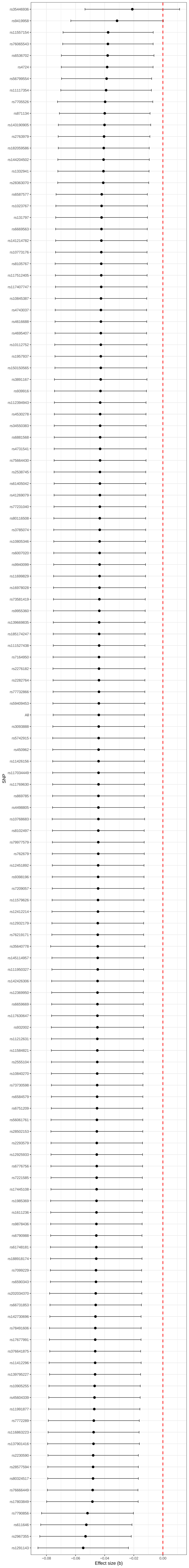

rs35446936
rs9419958
rs11557154
rs76065543
rs6536702
rs4724
rs56799554
rs11117354
rs7705526
rs871134
rs143190905
rs2763979
rs182059586
rs144204502
rs1332941
rs28363070
rs6587577
rs1023767
rs131797
rs6669563
rs141214782
rs10773176
rs8105767
rs117512405
rs117407747
rs10845387
rs4743037
rs4616688
rs4695407
rs10112752
rs1957937
rs150150565
rs3891167
rs939916
rs112394943
rs4530278
rs34550383
rs6881568
rs4731541
rs75664430
rs2538745
rs61405042
rs41269079
rs77231040
rs80116508
rs3785074
rs10805346
rs6007020
rs9940099
rs11699829
rs16978028
rs73581419
rs9955360
rs139669835
rs185174247
rs111527438
rs7164950
rs2276182
rs2282764
rs77732866
rs59409453
All
rs3093888
rs5742915
rs450962
rs11426156
rs117034449
rs11769630
rs869785
rs4498805
rs10768683
rs8102497
rs79977579
rs762679
rs12451892
rs9398196
rs7209057
rs11579626
rs12412214
rs12932179
rs76219171
rs35640778
rs145114957
rs111950327
rs142426306
rs12369950
rs6659669
rs117630647
rs932002
rs11212631
rs11584821
rs2555104
rs10840270
rs73730598
rs6584579
rs6751209
rs56061761
rs28502153
rs2293579
rs12925933
rs6776756
rs7221585
rs17445108
rs1985369
rs1611236
rs9878436
rs6790988
rs61748181
rs188918174
rs7099229
rs6590343
rs202034370
rs66731853
rs142730696
rs78491606
rs17677991
rs376641875
rs11412296
rs139795227
rs10905255
rs45604339
rs11991877
rs7772289
rs116863223
rs137901416
rs2230590
rs28577594
rs80324517
rs76666449
rs17803849
rs7790856
rs611646
rs2967355
rs1291143
SNP
−0.08
−0.06
−0.04
−0.02
0.00
Effect size (b)
